# Opioids and older adults: Increasing trends in opioid usage in a dental population compared to a National Database (NHANES)

**DOI:** 10.1111/scd.12709

**Published:** 2022-03-13

**Authors:** Piedad Suarez‐Durall, Maile S. Osborne, Chan Chan, Reyes Enciso, Roseann Mulligan

**Affiliations:** ^1^ Department of Geriatrics, Special Needs and Behavioral Sciences Herman Ostrow School of Dentistry, University of Southern California Los Angeles CA USA; ^2^ Leonard Davis School of Gerontology of University of Southern California Los Angeles CA USA; ^3^ Dental Public Health & Community Outreach Herman Ostrow School of Dentistry of University of Southern California Los Angeles CA USA

**Keywords:** gerodontology, NHANES, older adults, opioids, prevalence, self‐reported data

## Abstract

**Aim:**

To examine self‐reported opioid prevalence at a dental school clinic for patients ≥65 years old as compared to national data, comparing gender, ethnicity/race and older adult age groupings.

**Methods and results:**

Self‐reported prescription opioid medication use was extracted from the medical record for dental patients ≥65 years old who visited the school's general dental clinic (GDC) in 2012 or 2017. This data was compared to the National Health and Nutrition Examination Survey (NHANES) data for 2011–2012 and 2017–18. There was a significant increase in prevalence of opioid use in adults ≥65 between 2012 (4.5%) and 2017 (6.5%) and for ages 65–79 (from 4.7% to 6.3%) and ≥80 (3.4% to 7.9%), women (4.8% to 7.0%), and African Americans (4.7% to 8.4%) in the GDC. Older adults at the GDC reported less opioid use than the NHANES national average for both periods no matter the gender or the age with variable results for race/ethnicity.

**Conclusion:**

The prevalence of older adults taking opioids in our general dental school clinic population increased significantly in 2017 as compared to 2012 but was lower than the national average for the respective periods. Awareness of existing opioid usage in older adult patients and its higher adverse risk potential is critical when prescribing analgesics for dental pain for this age group.

## 1 INTRODUCTION

Although the rates of opioid involved deaths during 2017–2018 decreased in people aged 15–64, they increased among individuals aged 65 years and older.^1^ Opioid prescribing in the United States has increased since the 1990s, although the Centers for Disease Control and Prevention (CDC) indicates that the amount of pain reported by Americans overall has not changed.^2^ Aging, however, is often accompanied with increases in chronic pain; 30.8% of older Americans suffer from this condition,^3^ which is associated with prescription drug dependence^4^ and increased risk of injury.^5^ Data from the National Health and Nutrition Examination Survey (NHANES 2013–2016) shows this age discrepancy. While there is a 9.6% prevalence rate of prescription opioid use in the last 30 days for those ≥60 years old, substantially lower rates of 3.2% are found among adults aged 20–39 and 7.5% among those aged 40–59.^6^

Many government programs and public health campaigns such as the CDC's “Rx Awareness” emphasize the need to decrease opioid prescription usage in younger/middle aged adults (i.e., 25–54 years old) who have taken opioids at least once.^7^ Yet in 2018 alone, 25% of Americans ≥65 years old filled at least one opioid prescription^8^; with the years between 2008 and 2018, demonstrating the least amount of reduction for this age group.^8^ Some national polls^9^ and publications^5^, ^10^, ^11^, ^12^, ^13^ have targeted the opioid use of older adults, but there has been no widespread public campaign despite a call for such by the American Association of Retired Persons’ (AARP) Public Policy Institute.^4^

Chronic pain is known to impact the mobility, overall functioning and ultimately independence of older adults.^14^ A recent systematic review of opioid use for non‐cancer related chronic pain was focused on the higher rate of adverse events (78%)^15^ that opioids caused (e.g., nausea, dizziness, constipation, and any adverse event) when compared to placebo use. Els et al.^15^ mentioned that the more dire consequences of opioid usage (e.g., addiction, respiratory depression, cognitive dysfunction, endocrine dysfunction, and sleep apnea and sleep‐disordered breathing) were not covered by the investigators of the studies reviewed, possibly because the studies were not of adequate duration for these issues to emerge. Recommendations for future research include randomized clinical trials of longer duration that would report all adverse effects related to opioid usage, consistent adverse event terminology related to opioids, and the full dosing amount so that future investigations can better determine the true risks of opioid treatment.

There are additional factors that negatively impact opioid use in patients ≥65 years of age including reduced renal function and medication clearance, increased susceptibility to opioid accumulation, a smaller therapeutic window of safety, the coexisting use of other medications that are likely to interact with opioids (e.g., benzodiazepines), and cognitive impairment often resulting in greater risks of medication errors and misuse.^16^ In 2018 alone, there were 1152 prescription opioid‐involved overdose deaths for adults aged ≥65 years.^1^ Individuals with histories of falls and/or fractures are especially in jeopardy due to potentiation of risk when opioids are used with sedatives.^5^, ^17^

The American Geriatrics Society (AGS) Beers Criteria for potentially inappropriate medication use in older adults (≥65) specifically alerts providers to the harmful interactions between opioids and other medications.^18^ Furthermore, the rate of visits to the Emergency Department (ED) for those ≥65 years old diagnosed with an opioid‐related condition demonstrated a 74.2% increase between 2010 and 2015 compared to a 17.4% increase for those with nonopioid‐related ED visits.^11^ Those aged ≥65 years with four or more chronic conditions made up 33% of opioid‐related ED visits compared to 15.7% in the nonopioid‐related ED visits.^11^

When it comes to best clinical practices, the CDC has created guidelines for primary care physicians providing chronic pain treatment for patients in outpatient settings by substituting nonpharmacological management methods and nonopioid pain relievers in order to reduce opioid use disorder, overdose, and death.^16^ It is incumbent upon the dentists who manage chronic pain to follow this guidance as well.

Dental pain and pain related to dental procedures often result in a prescription for an opioid.^19^ Dentists have been identified among the leading prescribers of opioids behind primary care physicians and internists.^20^ Recognizing this, the American Dental Association (ADA) released an interim statement on the use of opioids in the treatment of dental pain and an official “Policy on Opioid Prescribing”^21^ supporting statutory dosing limits and obligatory training on prescribing opioids and other controlled substances.^21^ Given that 65.6% of older adults are now seeking dental care at least annually,^22^ it is critical that dentists understand both the prevalence of opioid usage in this age group and the seriousness of age‐related adverse effects, short and long term that compound the use of opioids.

Clinical research into opioids includes the NIDCR's sponsorship of deimplementation research in an effort to decrease opioid prescribing by dentists.^19^ One strategy being investigated is the placement of a clinical decision making tool within the electronic health record so that evidence‐based recommendations on nonopioid medications would be provided when an analgesic is needed. Should this strategy demonstrate the desired effect, the tool will be made available to dental practitioners. Additionally, the National Dental Practice‐Based Research Network is working with the NIDCR in a study to learn the extent of the opioid knowledge base of dental practitioners and their decision‐making strategies when prescribing opioids.^19^

The significant negative impact of opioid usage compelled us to investigate its prevalence in older dental patients attending a general dental clinic (GDC) of a dental school. Retrospective electronic health records from two different timepoints, 2012 and 2017 were acquired to examine opioid usage patterns in this population by gender, race/ethnicity, and two age groupings 65–79, and ≥80 years old. A second objective was to compare and contrast the prevalence of our findings to the use of prescribed opioids found in the national NHANES data, matched as closely as possible to similar data collection periods.

## 2 MATERIALS AND METHODS

### 2.1 Data collection

Permission to conduct this retrospective study was obtained from the University's Institutional Review Board (blinded). Demographics (age, gender, race/ethnicity, and Denti‐cal (yes/no) as well as self‐reported prescription opioid medication usage was extracted from the electronic health record for patients ≥65 years from the dental school's GDC in either 2012 or 2017 (Supporting Information Appendix S1 contains the [data set]). Patients at the dental school were asked about their current medications including name, dosage, and indication by the dental student or the staff at every visit.

In the GDC, typically community dwelling adults with stable medical conditions categorized by the American Society of Anesthesiologists Classification (ASA) as medical type I or II are treated.^23^ Our selection of patients was confined to the GDC as in this clinic the patients most resemble the enrollees in the NHANES study.

### 2.2 NHANES data collection methodology

Opioid prevalence for the general US population of individuals aged ≥65 years was calculated based on data downloaded from the NHANES website for 2011–2012 for all ages (*N* = 9756 completed the interview)^24^ and 2017–2018 (N = 9254 completed the interview).^25^ During the household interview, participants were asked by individuals trained in the NHANES interview protocol if they had taken any medications (last 30 days) for which they needed a prescription and to show their medication containers. For each medication reported, the interviewer enters the product's complete name from the container into the computer system and the name is automatically matched to a prescription medication. If an exact or similar match does not exist the interviewer selects “drug not found in list.”

The NHANES data is representative of the noninstitutionalized US civilian population of all ages residing in all 50 states and Washington DC. Overall analyses of our study data and comparison to a national data set were constrained due to the restrictions placed on the NHANES data. For example, geographic data is restricted and not openly available to the public to protect the confidentiality of survey respondents. Anyone 80 years or above was assigned an age of “80 years or older” by NHANES without precise ages provided. The NHANES [data sets] are publicly available.^24^, ^25^

### 2.3 Statistical analyses

Demographics by gender and race for GDC 2012 and 2017 were compared using Chi‐square tests. Prevalence of self‐reported use of opioids by gender, race/ethnicity, and age (65–79 and ≥80) for GDC 2012 and 2017 were compared using Chi‐square tests, and odds ratios (ORs) with 95% confidence intervals (CIs). Other races included Asian, Alaskan, Native Americans, Pacific Islander, multiple races, and unknown. National opioids self‐reported utilization rate with 95% CI for survey error based on NHANES data from periods of 2011–2012 and 2017–2018 were calculated for people ≥65 years, by gender, race/ethnicity, and age (65–79 and ≥80 years old) using survey weights following NHANES recommendations.^26^ Unadjusted and adjusted OR by age, gender, and race/ethnicity were calculated with logistic regression analyses for the GDC and NHANES datasets for 2012 and 2017. All analyses were conducted by one author (blinded) using SAS software version 9.4 (TS1M6).

## 3 RESULTS

There were no significant differences between the proportions of 65–79 and ≥80 years old, or genders in those attending the GDC at either time point in the study. The increased representation of Hispanics in 2017 was significant as was the decreased proportion of “other races” category (Table 1). Medicaid enrollment significantly increased in 2017 from the GDC 2012 baseline (Table 1).

**TABLE 1:** Demographics of patients aged 65–79 years and ≥80 years who attended a general dental clinic (GDC) at a dental school in 2012 and 2017

	GDC 2012 (*N* = 1816)	GDC 2017 (*N* = 2103)	GDC 2012 vs. 2017*p*‐value Chi‐square test
**Age, *n* (%)**			*p* > .05
65 to 79 y	1522 (83.8%)	1775 (84.4%)	
≥80 y	294 (16.2%)	328 (15.6%)	
Total	1816 (100%)	2103 (100%)	
**Gender, *n* (%)**			*p* > .05
Male	799 (44.0%)	935 (44.5%)	
Female	1017 (56.0%)	1168 (55.5%)	
Total	1816 (100%)	2103 (100%)	
**Race/ethnicity, *n* (%)**			*p* < .01
African American	340 (18.7%)	428 (20.4%)	*p* > .05
Caucasian	397 (21.9%)	428 (20.4%)	*p* > .05
Hispanic	368 (20.3%)	528 (25.1%)	*p* < .01
Other races	711 (39.2%)	719 (34.2%)	*p* < .01
Total	1816 (100%)	2103 (100%)	
**Insurance enrollment, *n* (%)**			*p* < .01
Medicaid (Denti‐cal)	135 (7.4%)	609 (29.0%)	
No medicaid	1681 (92.6%)	1494 (71.0%)	
Total	1816 (100%)	2103 (100%)	

**FIGURE 1:**
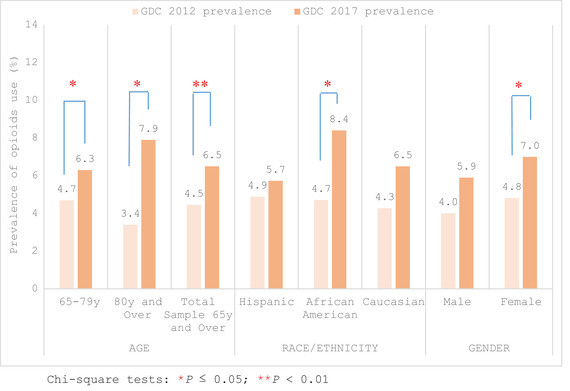
Prevalence of self‐reported opioid use in ≥65 years old in a general dental clinic (GDC) in 2012 (*n* = 1816) and 2017 (*n* = 2103) by age groups (65–79 y, ≥80 y), race/ethnicity, and gender. Chi‐square tests: *p ≤ .05; **p < .01

Prevalence of self‐reported opioid use for the GDC patients increased significantly for individuals ≥65 years old between 2012 (4.5%) and 2017 (6.5%) (Table 2, Figure 1). Patients aged 65–79 were nearly 1.4 times more likely to self‐report using opioids in 2017 (6.3%) than in 2012 (4.7%); with this rate increasing to nearly 2.5 times over the same time period for those aged 80 and over (3.4% in 2012 vs. 7.9% in 2017; Table 2). While there was an increase in prevalence of opioid use across all race/ethnicity categories in the GDC, it was only statistically significant in African American participants (4.7% in 2012 to 8.4% in 2017; Figure 1). Similarly, GDC opioid usage within each gender increased between 2012 and 2017, achieving statistical significance only in females (4.8% in 2012 vs. 7.0% in 2017; Figure 1, Table 2).

**TABLE 2:** Prevalence of self‐reported opioid use in ≥65 years old in a GDC in 2012 (*n* = 1816) and 2017 (*n* = 2103) by age groups (65–79 y, and ≥80 y), race/ethnicity, and gender with odds ratios

			GDC 2012	GDC 2017	OR			
Variable			Number of people (%)	Number of people (%)	Odds_2017/Odds_2012	95% Lower CI	95% Upper CI	*p*‐value Chi‐square
**Total sample**	≥65	Yes	81 (4.5%)	137 (6.5%)	1.49	1.13	1.98	<.01**
		No	1735 (95.5%)	1966 (93.5%)				
**Age (years)**	65–79	Yes	71 (4.7%)	111 (6.3%)	1.36	1.00	1.85	.05*
		No	1451 (95.3%)	1664 (93.8%)				
	≥80	Yes	10 (3.4%)	26 (7.9%)	2.45	1.16	5.16	.02*
		No	284 (96.6%)	302 (92.1%)				
**Gender**	Male	Yes	32 (4.0%)	55 (5.9%)	1.50	0.96	2.34	.07
		No	767 (96.0%)	880 (94.1%)				
	Female	Yes	49 (4.8%)	82 (7.0%)	1.49	1.04	2.15	.03*
		No	968 (95.2%)	1086 (93.0%)				
**Race/ethnicity**	Caucasian	Yes	17 (4.3%)	28 (6.5%)	1.56	0.84	2.91	.15
		No	380 (95.7%)	400 (93.5%)				
	African American	Yes	16 (4.7%)	36 (8.4%)	1.86	1.01	3.41	.04*
		No	324 (95.3%)	392 (91.6%)				
	Hispanic	Yes	18 (4.9%)	30 (5.7%)	1.17	0.64	2.13	.61
		No	350 (95.1%)	498 (94.3%)				

CI, confidence interval; GDC, general dental clinic; OR, odds ratio. Chi‐square tests * *p* ≤ .05; ** *p*< .01.

National opioids self‐reported utilization data for older adults aged ≥65 y from 2011–2012 (*N* = 1250) and 2017–2018 (*N* = 1500) was derived from NHANES data with the 2011–2012 dataset representing 36,881,034 older adults, and the 2017–2018 representing 45,186,952. [Correction added on 8 September 2022, after first online publication: the dataset values were changed]. There were no significant differences in opioid use of the NHANES participants between these two time periods by age, gender, or race/ethnicity (Table 3) with one exception. There was a significant decrease in Hispanics opioid use (*p* = .02).

**TABLE 3:** Prevalence of self‐reported opioid use in ≥65 years old in a GDC in 2012 (*n* = 1816) and 2017 (*n* = 2103) and NHANES 2011–2012 and 2017–2018 with 95% CI by age groups (65–79 and ≥80 y), race/ethnicity, and gender

				NHANES 2011–2012			
		GDC 2012				95% CI for mean	
Variable		*N*	Prev.	*N*	Prev.	Lower	Upper
**Total sample**	≥ 65	81	4.5%*	111	6.9%	4.6%	9.3%
**Age (years)**	65 to 79	71	4.66%*	88	7.7%	4.70%	10.6%
	≥80	10	3.4%	23	4.9%	2.4%	7.5%
**Gender**	Male	32	4.0%	49	6.3%	3.7%	8.9%
	Female	49	4.8%	62	7.4%	4.6%	10.3%
**Race/ethnicity**	Caucasian	17	4.3%	49	6.3%	3.2%	9.5%
	African American	16	4.7%*	36	12.2%	7.2%	17.1%
	Hispanic	18	4.9%	18	8.8%	4.4%	13.2%

Variable		GDC 2017		NHANES 2017–2018			
Overall	≥65	137	6.5%	110	8.0%	5.7%	10.2%
**Age**	65 to 79	111	6.3%	82	8.4%	6.0%	10.7%
	≥80	26	7.9%	28	6.7%	3.3%	10.1%
**Gender**	Male	55	5.9%	54	6.3%	3.9%	8.6%
	Female	82	7.0%	56	9.4%	5.5%	13.3%
**Race/ethnicity**	Caucasian	28	6.5%	61	8.7%	5.9%	11.5%
	African American	36	8.4%	29	9.0%	5.4%	12.6%
	Hispanic	30	5.7%	12	4.4%	2.2%	6.7%

CI, confidence interval; GDC, general dental clinic; NHANES, National Health and Nutrition Examination Survey. ^*^ The GDC prevalence rate estimate was outside the 95% CI of the estimated prevalence for NHANES data.

**FIGURE 2:**
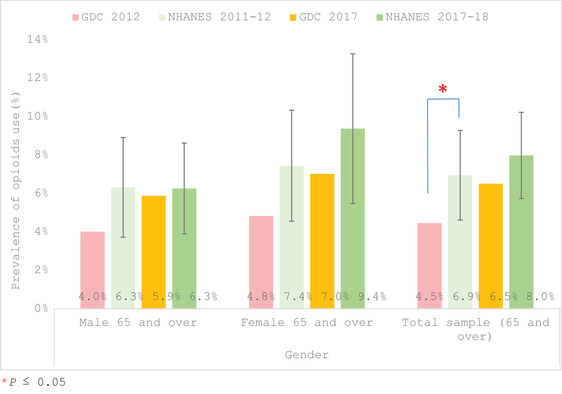
Prevalence of self‐reported use of at least one opioid by gender and overall for individuals aged ≥65 years old at the GDC in 2012 and 2017 in comparison to NHANES 2011–2012 and 2017–2018. Error bars show the 95% CI of the estimated prevalence for NHANES data. * *p* ≤ .05. CI, confidence interval; GDC, general dental clinic; NHANES, National Health and Nutrition Examination Survey

**FIGURE 3:**
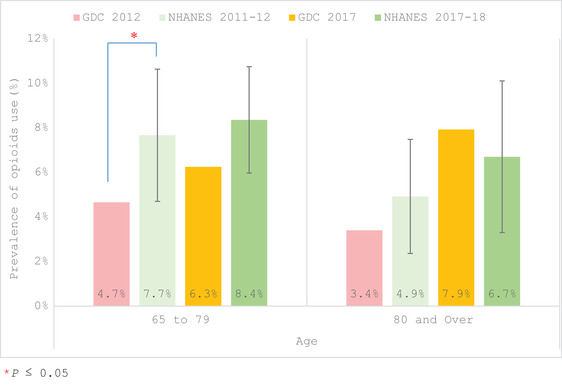
Prevalence of self‐reported use by age of at least one opioid for individuals aged ≥65 years old at the GDC in 2012 and 2017 in comparison to NHANES 2011–2012 and 2017–2018. *p ≤ .05. GDC, general dental clinic; NHANES, National Health and Nutrition Examination Survey

**FIGURE 4:**
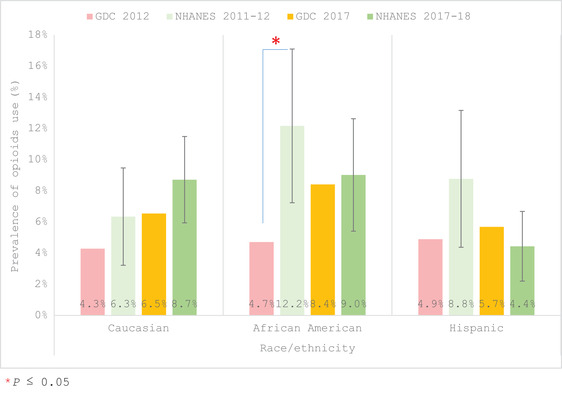
Prevalence of self‐reported use by race/ethnicity of at least one opioid for individuals aged ≥65 years old at the GDC in 2012 and 2017 in comparison to NHANES 2011–2012 and 2017–2018. Error bars show the 95% CI of the estimated prevalence for NHANES data. * *p* ≤ .05. CI, confidence interval; GDC, general dental clinic; NHANES, National Health and Nutrition Examination Survey

In comparing the 2012 GDC older adults to the NHANES 2011–2012, the GDC patients reported a significantly lower prevalence of opioids than the NHANES (4.5% in GDC vs. 6.9%; Table 3, Figure 2). This was also true of those 65–79 years old with a prevalence of 4.66% in the GDC versus 7.7% for the NHANES (Table 3, Figure 3). Overall, the trend for the GDC patients was to self‐report less opioid use than the NHANES population for the respective time periods (2012 and 2017), no matter the gender (Figure 2) or age (Figure 3), with the exception of nonsignificant trends for those aged ≥80 and Hispanics who reported higher usage in the GDC 2017 than in the NHANES 2017–2018. When reviewing the race/ethnicity outcomes (Figure 4) a significant difference in prevalence was only seen in African Americans with opioid usage for GDC participants being 4.7% versus 12.2% for the 2011–2012 NHANES (Table 3, Figure 4).

Of those patients taking opioids seen at the GDC in 2012, 14.8% had Dentical insurance compared to 29.9% in 2017. In contrast, of those patients taking opioids in the national representative dataset, the percentage of patients enrolled in Medicaid decreased from 15.1% in 2012 to 11.6% in 2017.

Further analysis using logistic regression demonstrated the adjusted ORs for opioid use were not statistically significantly different for males versus females, 65–79 y versus ≥80 year‐olds or ethnicity/race in 2012 (Supporting Information Table S2) and 2017 (Supporting Information Table S3) for either GDC nor NHANES data; except for the NHANES 2017–2018 data in which Hispanics reported about half the use of opioids than Caucasians (adjusted OR = 0.468; 95% CI = 0.270–0.808; Supporting Information Table S3).

## 4 DISCUSSION

Despite public health campaigns against overprescribing opioid drugs and increased federal and state regulation of prescribers of opioids, our data demonstrated an overall significant increase in the prevalence of self‐reported opioid usage by GDC patients aged ≥65 years between 2012 (4.5%) and 2017 (6.5%), and in both age categories (65–79 and over 80 years old). This is consistent with annualized data published by the California Department of Public Health demonstrating increasing opioid usage in all older adult age groups (65–74, 75–79, 80—84, and 85+ years old).^27^ We also found significant increases in females and African Americans using opioids (Figure 1). During this same time period the Los Angeles (LA) County Department of Public Health, which covers the area where our school clinic is located, recorded an increased number of treatment admissions for African American women in which they cited prescription opioids as their primary drug problem.^28^

Using the Medicare Current Beneficiary Survey data, Skaar and O'Connor^29^ not only found that 30.38% of older adults had received at least one opioid prescription during dental treatment, there was a higher prevalence of women and non‐whites being prescribed potentially inappropriate medications (PIMs) as identified using the 2015 Beers Criteria. Additionally Zhou et al.^30^ showed that older females, especially those with pre‐existing mental health issues including mood disorders and psychoses are often treated with PIMs. Prescribing opioids for them, even for short term acute dental pain, carries significant risk for a subsequent ED visit or hospital admission.

Our overall lower findings of opioid use in the dental school's GDC compared to the national average in 2017 demonstrates the geographic differences in opioid dispensing rates evident across the US^31^ and is consistent with opioid prescription rates being lower in LA County (30.3 per 100 people) compared to the US average of 46.7 prescriptions per 100 persons reported by the CDC.^31^

Older adults are retaining more teeth (an average of 21 as of 2016),^32^ and seeking dental care in greater numbers.^22^ Therefore, the likelihood for more extractions and other surgical procedures looms. Dentists need to be aware of the increasing profile of opioid usage by older adult dental patients for chronic pain conditions upon presentation to the dental office and consider the potential for abuse, misuse, and side effects when planning post‐treatment analgesic support. Data demonstrates that in 2019, 22.3% of US dental prescriptions were written for opioids, yet there were only 0.6% written in England, where NSAIDs and acetaminophen accounted for most analgesic prescriptions.^33^ In support of this analgesic direction, Moore et al.^34^ highlighted the use of 400 mg of ibuprofen with 1000 mg of acetaminophen as a medication combination superior to any opioid‐containing medication with a decreased risk of inducing acute adverse events.

Prescription drug monitoring programs (PDMP) are mandatory in many states and have been shown to reduce the number of prescribed opioids significantly, for example, in New York state, there was a 78% decrease in the absolute number of opioid pills prescribed in a 3‐month period after such a program was instituted.^35^ Zhou et al.^30^ mentioned that the limited access to medication reconciliation available to dentists increases the risk for adverse reactions in older adults taking opioids. Further they suggest that dentists may need support from a pharmacist to have a more comprehensive medication reconciliation program for the patients. Tools such as STOPP (Screening Tool of Older Persons Prescriptions) and START (Screening Tool to Alert to Right Treatment) could assist the dentist in identifying medications with adverse drug events.^29^

Additionally, dentists should be aware of CDC guidelines,^16^ ADA policies^21^ as well as the Beers Criteria^18^ not only for writing their own prescriptions but to better educate their patients about various opioids risks and issues with opioid medications they may be taking for non‐dental therapy. For example, CDC guidelines^16^ recommend educating older adults on avoiding controlled medications from multiple prescribers and on how to safely dispose of unused opioids.

Opioid adverse events and side effects cannot be overstated. Many of these consequences such as endocrinopathies,^36^ respiratory depression,^37^ dry mouth,^15^ periodontal disease, and caries, put older adults at higher risk of frailty.^38^ Opioid‐induced endocrinopathies, including hypogonadism and the inhibitory effects of opioid drugs on the hypothalamo‐pituitary‐adrenal axis require special attention as they can result in negative effects on muscle mass and bone health and the increased risk of fracture, attributed to high risk of falls, due to the dizziness and sedation from opioids.^36^

The interaction of poor oral health, chronic pain, and opioid usage as potential risk factors for frailty need further study as they can have immediate clinical implications (e.g., opioid‐induced adverse health‐related outcomes including delirium, forgetting, falls, constipation, unintentional, and intentional overdose) as well as those that are multi‐dimensional (e.g., cognitive decline, dementia, malnutrition, sarcopenia, falls, infection, depression, weak cough, and aspiration risk).^38^ Ultimately, patients with chronic pain using opioids are at higher risk for suicide attempts or intentional overdoses^39^; suicides could be undercounted or misclassified as opioid‐related poisoning deaths.^39^

Improving oral health as a contributor of frailty will require a great deal of effort. According to Chalmers 2005,^40^ as the existing geriatric dental trained workforce cannot meet the existing needs, nurses, and other care providers in long‐term facilities need to be trained to assess oral needs and refer to the dentist as appropriate. Assessment of oral health in older adults by non‐dental professional caregivers requires intensive training.^41^ In one pilot study with four nurses, an intensive training of 12 hours resulted in high agreements on oral care plans with an oral health professional.^42^ The validity and reliability of present oral health assessment tools especially when used by non‐oral health professionals need to be further researched.^43^ The World Dental Federation is also working on creating an oral health assessment to be used by non‐dental healthcare professionals.^43^

### 4.1 Strengths and limitations

A strength of this study was the inclusion of a national dataset surveying the prevalence of opioid use by older adults compared with those seeking care from a local dental school clinic. A further strength was that the sample size of the local and national datasets did allow many comparisons and statistical analyses by age, gender, and race/ethnicity. There were also some limitations. For example, it is known that self‐reporting of medication usage collected in both data sets when provided by older adults could be questionable.^44^ Whereas the NHANES’ data allowed for additional contemporaneous verification of prescription medications, our retrospective analysis of the GDC EHR did not support this strategy. While we agree that some older adults may be forgetful of some of their prescribed medications, we also appreciate that, borrowing medications from others is minimal (less than 10%) [30], as is obtaining opioids from illicit sources.^12^

It should be noted that the NHANES study does collect annual household income and insurance data including Medicaid enrollment, however the GDC only collected Medicaid enrollment. Therefore, we used Medicaid enrollment as a proxy for low income. In the NHANES 2011–2012 and its matching GDC dataset the proportion of older adults receiving Medicaid were similar (Supporting Information Table S1). This was not true of the NHANES 2017–2018 period versus GDC 2012, as Medicaid eligibility for adults specifically living in the State of California significantly expanded beginning in 2014 and years following with its enrollment far outstripping that of the NHANES 2017–2018 period (Supporting Information Table S1).^45^

Research on opioid usage in older adults is a vitally important area of study due to the present lack of attention it currently receives and the major impact it can have on the individual's life. With this analysis of opioid data usage locally and nationally as a foundation, we look forward to ongoing studies that continue to examine changes in opioid usage in older adults. Although the next NHANES 2019–2020 data set has yet to be released, we recommend that other datasets that may be available be reviewed if we are to make progress on issues related to opioid use by older adults.

## 5 CONCLUSION

In summary, this study's particular goal was to compare the prevalence of opioid usage in older adults who were already taking opioid medications upon presentation to the dental clinic and compare those findings to a national database. We learned that the prevalence of older adult opioid users in our general dentistry clinic was less than the national average, demonstrating an increase in prevalence between 2012 and 2017 with some similarities and differences by age, gender, and race/ethnicity compared to NHANES data. The rates of opioid usage found highlight the need to increase awareness of the CDC guidelines, ADA policy, and the Beers Criteria and the crucial need for continuing education about the risks of prescribing opioids versus alternative dental pain reduction modalities. Opioid usage rates vary by geography and there are many helpful CDC websites and maps that provide information down to the county level on opioid dispensing, ED visit rates and death rates that could better inform the dentist of the potential opioid profile of the community and the patient risk in his/her practice. We recommend that all dental clinicians take thorough medication histories and become aware of the increased risk to older adults who are already taking opioid medications for other reasons when visiting the dental clinic. We also recommend that dentists investigate alternative non‐opioid pain relief approaches should analgesics become necessary to increase patient comfort like patient education, counseling, and behavior modification methods.

## ETHICS STATEMENT

Permission to conduct this research was submitted to and approved by the University Park Institutional Review Board of the University of Southern California. The study qualified as Exempt with title “Profile of Older Patients Seeking Dental Care (Retrospective)” and number IRB #UP‐12‐00232.

## AUTHOR CONTRIBUTION

All authors have made (1) substantial contributions to conception and design of, or acquisition of data or analysis and interpretation of data, (2) drafting the article or revising it critically for important intellectual content, and (3) final approval of the version to be published.

## CONFLICT OF INTEREST

The authors declare that they have no conflict of interest.

## Supporting information

Supporting Information Table

Supporting Information Table S1
Supporting Information Table S2
Supporting Information Table S3
