# Supplementary material for: Opioids and older adults: Increasing trends in opioid usage in a dental population compared to a National Database (NHANES)
Source: Spec Care Dentist. 2022 Mar 13;42(5):445–55. doi: 10.1111/scd.12709 (PMC9543923; doi:10.1111/scd.12709)
Supplement: Supplementary file 2 — Supporting Information Table S1 Supporting Information Table S2 Supporting Information Table S3 [file SCD-42-445-s002.docx]

**Supporting Information Table S1.** M*edicaid enrollment and opioid usage in a general dental clinic (GDC) in 2012 (n=1,816) and 2017 (n=2,103) and NHANES 2011-12 and 2017-18.*

| Opioids usage | **GDC 2012** | | | | | **GDC 2017** | | | | |
| --- | --- | --- | --- | --- | --- | --- | --- | --- | --- | --- |
|  | Insurance | | | Total | | Insurance | | | Total | |
|  | Denti-Cal,  n (%) | No Dentical, n (%) |  | | Denti-Cal,  n (%) | | No Dentical,  n (%) |  | |  |
| Yes | 12 | 69 | 81 | | 41 | | 96 | 137 | |  |
|  | 14.8% | 85.2% | 100.0% | | 29.9% | | 70.1% | 100.0% | |  |
| No | 123 | 1612 | 1735 | | 568 | | 1398 | 1966 | |  |
|  | 7.1% | 92.9% | 100.0% | | 28.9% | | 71.1% | 100.0% | |  |
| Total | 135 | 1681 | 1816 | | 609 | | 1494 | 2103 | |  |
|  | 7.4% | 92.6% | 100.0% | | 29.0% | | 71.0% | 100.0% | |  |
|  |  |  |  | |  | |  |  | |  |
| Opioids usage | **NHANES 2011-2012** | | | | | **NHANES 2017-2018** | | | | |
|  | Insurance† | | | Total | | Insurance† | | | Total | |
|  | Medicaid,  n (%) | No Medicaid,  n (%) |  | | Medicaid,  n (%) | | No Medicaid,  n (%) |  | |  |
| Yes | 24 | 87 | 111 | | 28 | | 82 | 110 | |  |
|  | 15.1% | 84.9% | 100.0% | | 11.6% | | 88.4% | 100.0% | |  |
| No | 131 | 1008 | 1139 | | 169 | | 1221 | 1390 | |  |
|  | 6.3% | 93.7% | 100.0% | | 6.4% | | 93.6% | 100.0% | |  |
| Total | 155 | 1095 | 1250 | | 197 | | 1303 | 1500 | |  |
|  | 6.9% | 93.1% | 100.0% | | 6.8% | | 93.2% | 100.0% | |  |

† **Percentages are weighted**

**Supporting Information Table S2.** *Unadjusted and adjusted Odds Ratios for opioid self-reported use at GDC 2012 and NHANES in 2011-12 for ≥65 years old.*

| Variable | **GDC 2012** | | | | **NHANES 2011-12** | | | | |
| --- | --- | --- | --- | --- | --- | --- | --- | --- | --- |
|  | Unadjusted Model | | Adjusted  Model† | | Unadjusted  Model | | | Adjusted  Model† | |
|  | OR | 95% CI | OR | 95% CI | OR | 95% CI | OR | | 95% CI |
| 65-79 | 1.000 | -- | 1.000 | -- | 1.000 | -- | 1.000 | | -- |
| ≥80 | 0.720 | 0.367-1.413 | 0.729 | 0.371-1.432 | 0.623 | 0.317-1.227 | 0.630 | | 0.306- 1.299 |
| Male | 1.000 | -- | 1.000 | -- | 1.000 | -- | 1.000 | | -- |
| Female | 1.213 | 0.769-1.913 | 1.188 | 0.750-1.882 | 1.192 | 0.748-1.901 | 1.218 | | 0.756-1.964 |
| Caucasian | 1.000 | -- | 1.000 | -- | 1.000 | -- | 1.000 | | -- |
| African American | 1.104 | 0.549-2.220 | 1.087 | 0.540-2.191 | 2.047 | 0.932-4.494 | 1.957 | | 0.867-4.419 |
| Hispanic | 1.150 | 0.583-2.266 | 1.103 | 0.556-2.187 | 1.420 | 0.691-2.917 | 1.371 | | 0.658-2.856 |

† Adjusted for age categories (65-79, ≥80), gender (male/female) and race/ethnicity (Caucasian, African American and Hispanic).

**Supporting Information Table S3.** *Unadjusted and adjusted Odds Ratios for opioid self-reported use at GDC 2017 and NHANES in 2017-18 for ≥65 years old.*

| Variable | **GDC 2017** | | | | **NHANES 2017-18** | | | |
| --- | --- | --- | --- | --- | --- | --- | --- | --- |
|  | Unadjusted  Model | | Adjusted Model† | | Unadjusted  Model | | Adjusted  Model† | |
|  | OR | 95% CI | OR | 95% CI | OR | 95% CI | OR | 95% CI |
| 65-79 | 1.000 | -- | 1.000 | -- | 1.000 | -- | 1.000 | -- |
| ≥80 | 1.291 | 0.828-2.013 | 1.253 | 0.801-1.959 | 0.788 | 0.468-1.328 | 0.742 | 0.427-1.291 |
| Male | 1.000 | -- | 1.000 | -- | 1.000 | -- | 1.000 | -- |
| Female | 1.208 | 0.849-1.719 | 1.223 | 0.855-1.751 | 1.550 | 0.801-2.998 | 1.571 | 0.796-3.101 |
| Caucasian | 1.000 | -- | 1.000 | -- | 1.000 | -- | 1.000 | -- |
| African American | 1.312 | 0.785-2.192 | 1.249 | 0.744-2.096 | 1.039 | 0.632-1.710 | 1.014 | 0.604-1.701 |
| Hispanic | 0.861 | 0.506-1.464 | 0.825 | 0.481-1.415 | **0.487*** | **0.286-0.828*** | **0.468*** | **0.270-0.808*** |

† Adjusted for age categories (65-79, ≥80), gender (male/female) and race/ethnicity (Caucasian, African American and Hispanic).

* The 95% CI does not include OR=1, therefore the result is statistically significant.
